# Depressive cognitions as a mediator between adverse childhood experiences, resilience, and depression in Taiwanese university students

**DOI:** 10.1080/00049530.2025.2609408

**Published:** 2026-02-01

**Authors:** Shin-Yi Lu, Ya-Ting Juang, Cheng-Liang Hsu

**Affiliations:** a Office of Student Affairs, National Taipei University of Business, Taipei City, Taiwan; b Department of Psychology and Counseling, University of Taipei, Taipei City, Taiwan; c Department of Electrical Engineering, National University of Tainan, Tainan City, Taiwan

**Keywords:** Adverse childhood experiences, depressive cognitions, depressive symptoms, resilience, university students

## Abstract

**Objective:**

Adverse childhood experiences (ACEs), resilience, and depressive cognitions are recognised predictors of depressive symptoms. However, limited evidence exists on how depressive cognitions mediate these relationships in university students, a population facing developmental and psychological challenges during emerging adulthood. This study aimed to examine the indirect effects of depressive cognitions between ACEs, resilience, and depressive symptoms.

**Method:**

A cross-sectional survey was conducted with 552 university students (aged 20–23) from five universities in northern Taiwan. Participants completed validated self-report measures assessing ACEs, trait resilience, depressive cognitions, and depressive symptoms. Structural equation modelling (SEM) was employed to test the hypothesised model, and bootstrapping with 1000 resamples was used to assess indirect effects.

**Results:**

ACEs were positively associated with depressive cognitions and depressive symptoms, while resilience was negatively associated with both. Moreover, depressive cognitions significantly accounted for indirect pathways between ACEs, resilience, and depressive symptoms.

**Conclusions:**

These findings highlight depressive cognitions as a key psychological process through which early adverse experiences and trait resilience are associated with depressive outcomes. Targeted interventions focusing on cognitive restructuring may help reduce depressive symptoms among university students, given the central role of depressive cognitions observed in this study.

## KEY POINTS

**What is already known about this topic:**

1. ACEs are linked to greater risk of depressive symptoms during emerging adulthood.
2. Resilience is a known protective factor associated with lower depressive symptoms.
3. Cognitive patterns play a significant role in the development and maintenance of depressive symptoms.

**What this topic adds:**

1. This study examines how depressive cognitions serve as a psychological pathway linking ACEs and resilience to depressive symptoms.
2. Findings are based on a Taiwanese university student sample, contributing to non-Western evidence.
3. The results underscore the potential value of early cognitive-focused approaches for addressing trauma-related depressive symptoms.

## Introduction

Adverse childhood experiences (ACEs) refer to significant stressors or traumatic events occurring before the age of 18, encompassing both traditional forms of adversity such as abuse, neglect, and household dysfunction (Felitti et al., 1998, 2019), and expanded forms of adversity occurring outside the home environment, including bullying, community violence, discrimination, or poverty (Perrins et al., 2024; World Health Organization, n.d.). These experiences have profound and enduring effects on psychological development and have been consistently linked to various mental health problems in adulthood, including depressive symptoms, anxiety, and post-traumatic stress disorder (PTSD) (Felitti et al., 1998, 2019). However, the specific cognitive mechanisms through which early adversity contributes to depressive outcomes remain insufficiently understood.

Although the effects of ACEs have been widely examined among children and adolescents (Madigan et al., 2025) and adults (Madigan et al., 2023), an increasing number of studies have recently begun to focus on university students. This population typically represents the stage of emerging adulthood, a developmental phase situated between adolescence and full adulthood (Akter et al., 2025; Albers et al., 2022; Arnett, 2014). Emerging adulthood is a critical yet relatively understudied period characterised by identity exploration, increasing independence, and emotional regulation challenges, often accompanied by reduced external support. Empirical evidence indicates that university students with ACE histories are at greater risk for depression, suicidality, and psychological distress compared with their peers without such experiences (Calegaro et al., 2023; Muwanguzi et al., 2023; Raghunathan et al., 2024). Yet, little is known about the psychological pathways that explain how ACEs contribute to depressive symptoms in this population, particularly within non-Western contexts where empirical data remain limited.

Individuals with high resilience typically exhibit personal characteristics such as optimism, perseverance, commitment, and adaptability (Connor & Davidson, 2003), and this trait-like resilience has been shown to be associated with lower levels of depressive symptoms (Diehl et al., 2022; Ying et al., 2014). Nevertheless, the cognitive mechanisms underlying this protective association remain largely unexplored. On the other hand, based on Beck’s cognitive theory of depression, negative beliefs about the self, the world, and the future – known as the depressive cognitions – contribute to the onset and maintenance of depressive symptoms (Beck et al., 1979).

Accordingly, the present study focuses on examining the psychological pathways linking ACEs, depressive cognitions, and depressive symptoms, while also exploring the psychological mechanisms connecting resilience, depressive cognitions, and depressive symptoms. By integrating these risk and protective processes, this study seeks to clarify how ACEs and resilience are independently related to depressive symptoms through depressive cognitions during emerging adulthood.

To achieve these aims, the study adopted a parallel mediation framework, conceptualising ACEs as an independent risk predictor—(1) directly associated with higher depressive symptoms, and (2) indirectly associated with depressive symptoms through increased depressive cognitions. Meanwhile, resilience was modelled as an independent protective predictor—(1) directly associated with lower depressive symptoms, and (2) indirectly associated with depressive symptoms through reduced depressive cognitions. Through this conceptual framework, the present study seeks to deepen the understanding of how ACEs, resilience, and depressive cognitions relate to depressive symptoms and shape mental health during emerging adulthood.

### Developmental context of university students

University students, typically in the stage of emerging adulthood, occupy a unique developmental phase between adolescence and full adulthood (Arnett, 2014). During this stage – generally spanning from the late teens to the twenties – individuals experience major life transitions characterised by identity exploration, increasing autonomy, and instability across academic, social, and financial domains (Wood et al., 2017; Wider et al., 2023). Compared with adolescents who benefit from parental and institutional support, and adults who have generally developed mature coping strategies, university students must manage multiple stressors independently (Arnett, 2000; Schulenberg & Maggs, 2002; Wood et al., 2017). Although many develop new coping capacities, they may still be forming the psychological maturity characteristic of older adults, making them more vulnerable to stress and emotional challenges.

For students with a history of adverse childhood experiences (ACEs), these challenges can be particularly pronounced. Prior trauma may compromise emotional regulation and adaptive functioning, heightening the risk for psychological distress (Bartolomé-Valenzuela et al., 2024; Craig et al., 2023). Bhattarai et al. (2023) found that, in a longitudinal cohort of first-year university students, those reporting any childhood abuse (physical or sexual abuse or peer bullying) were more likely than those reporting none to screen positive for common mental health problems, including depression, anxiety, self-harm, and suicidality. Similarly, Watt et al. (2022) found that college students with four or more ACEs were significantly more likely than those with fewer ACEs to report depressive and anxiety symptoms.

Across developmental stages, the impact of ACEs may differ depending on emotional and contextual resources (Herzog & Schmahl, 2018). Adolescents exposed to ACEs may benefit from parental monitoring and structured educational settings that buffer against harm (Lynne et al., 2025; Wang et al., 2021), whereas adults tend to rely on greater emotional maturity and self-regulation to manage adversity (Ryan & Deci, 2000). In contrast, emerging adults – particularly university students – often face complex transitions without consistent external scaffolding, leaving them more susceptible to psychological distress. Such vulnerabilities are especially evident among those with ACE histories, which have been linked to poorer psychological adjustment, greater emotional dysregulation, and lower health-related quality of life in emerging adulthood (Cohrdes & Mauz, 2020; Feiler et al., 2023; Hazzard et al., 2021).

Together, these developmental characteristics underscore the importance of examining ACE-related mental health outcomes among university students, who are navigating the challenges of emerging adulthood.

### Theoretical framework: parallel mediation effects of ACEs and resilience on depressive symptoms through depressive cognitions

#### Adverse childhood experiences and depressive symptoms

ACEs have been widely recognised as potent predictors of depressive symptoms across the lifespan (Desch et al., 2023). Research consistently demonstrates that individuals with greater ACE exposure tend to report higher levels of depressive symptoms, a pattern observed across diverse cultures and age groups. For example, studies among university students in Vietnam and Korea revealed that exposure to three or more ACE categories was associated with increased depressive symptom severity, reduced well-being, and heightened suicidal ideation (Kim, 2017; Tran et al., 2015). Similar dose – response relationships have been reported in adult and older populations in China, Ireland, and Uganda, where each additional ACE was linked to greater depressive symptom risk or intensity (Chang et al., 2019; Chen et al., 2022; Cheong et al., 2017; Satinsky et al., 2021).

A meta-analysis of over 190 studies further confirmed the strong associations between childhood maltreatment – particularly emotional abuse and neglect – and both clinical depression diagnoses and depressive symptom levels (Humphreys et al., 2020). Longitudinal evidence has also shown consistent associations, indicating that early adversity is related to persistent depressive trajectories and heightened vulnerability in adulthood (Peng & Liang, 2023; Yin et al., 2024). Together, these findings underscore the cumulative and enduring associations between ACEs and mental health outcomes.

#### Depressive cognitions as a psychological mechanism

Depressive cognitions have been widely recognised as a key psychological correlate of emotional distress associated with early adversity. According to Beck’s Cognitive Theory of Depression, negative beliefs about the self, the world, and the future – known as the cognitive triad – contribute to the onset and maintenance of depressive symptoms (Beck et al., 1979). Childhood adversity may be linked to the internalisation of maladaptive schemas, including feelings of helplessness, worthlessness, and hopelessness, as well as perceptions that the world is uncontrollable or unsafe (Korotana et al., 2016). Empirical studies support these associations. Briere and Elliott (1994) found that survivors of childhood abuse often reported cognitive distortions such as low self-worth, chronic mistrust, and a sense of powerlessness, which persist into adulthood. Similarly, Poletti et al. (2014) reported a positive association between ACE severity and cognitive distortions, particularly overgeneralised beliefs of uncontrollability across contexts. Emotional maltreatment, more so than physical abuse, has been associated with the development of negative cognitive styles (Gibb, 2002). Longitudinal studies have also shown that peer victimisation in childhood is related to increases in depressive cognitions over time, including automatic thoughts of social threat and negative perceptions of the self, world, and future (Sinclair et al., 2012).

Furthermore, studies among children and adolescents have consistently shown that more negative cognitive triad scores are associated with greater depressive symptoms (Braet et al., 2015; Jacobs & Joseph, 1997; Stark et al., 1996). These findings suggest that depressive cognitions may help explain associations between ACEs and depressive symptoms (Briere & Elliott, 1994; Korotana et al., 2016), and may serve as early psychological indicators of depressive vulnerability (Beck, 1987; Guo et al., 2017). Taken together, the evidence supports a framework in which childhood adversity is associated with the development of depressive cognitions – negative perceptions of the self, world, and future – that, in turn, are linked with elevated depressive symptom levels.

#### Resilience and depressive symptoms

Resilience is conceptualised in this study as a relatively stable personality trait that reflects an individual’s capacity to manage adversity with flexibility and persistence (Connor & Davidson, 2003). Individuals with high trait resilience tend to exhibit greater self-efficacy, emotional regulation, and tolerance for distress, which are often associated with lower levels of depressive symptoms.

Empirical studies consistently show that higher resilience is associated with lower levels of depressive symptoms across different populations, including adolescents, university students, and older adults (Färber & Rosendahl, 2020; Ran et al., 2020; Shebuski et al., 2020; Ying et al., 2014). Longitudinal findings have also demonstrated consistent associations, suggesting that trait resilience may help mitigate depressive symptoms over time, even under prolonged stress (Wu et al., 2020). Collectively, these findings highlight the potential protective role of resilience in mental health, particularly in relation to the psychological impact of early adversity and ongoing stressors.

#### Resilience and depressive cognitions

While resilience has been widely discussed as a protective factor associated with lower levels of depressive symptoms (Färber & Rosendahl, 2020; Tamura et al., 2021), empirical evidence also supports its cognitive correlates. Specifically, resilience has been linked to more adaptive thinking patterns and fewer negative cognitive biases – features that align closely with Beck’s cognitive model of depression (Beck, 1987; Beck et al., 1979).

For instance, Stapleton et al. (2017) found that higher resilience among adolescents was associated with a lower fear of failure, reflecting reduced negative expectations about the future. Similarly, Hernandez et al. (2016) reported that individuals with greater resilience expressed fewer stigma-related beliefs towards mental health help-seeking – indicating less distrust and threat-based thinking about the social world. In a large university sample, Mak et al. (2011) demonstrated that trait resilience was positively associated with more favourable views of the self, world, and future, and indirectly reduced depression through these positive cognitions. Hatami et al. (2019) further found that resilience was positively correlated with hope, an indicator of future-oriented optimism. More recently, Li and Zheng (2025) also showed that individuals with higher trait resilience tend to hold stronger beliefs in their ability to manage adversity and maintain positive expectations about the future.

Collectively, these findings suggest that resilience may foster adaptive cognitive appraisals that counteract the development of depressive cognitions – maladaptive thought patterns involving hopelessness, self-devaluation, and negative worldview. In this sense, trait resilience may exert an indirect influence on depressive symptoms through its association with depressive cognitions. Consistent with trait-based conceptualisations of resilience (Connor & Davidson, 2003; Wagnild & Young, 1993), this study treats resilience as a relatively stable psychological trait that supports positive adaptation. In the present model, ACEs and trait resilience are conceptualised as independent domains of risk and protection, each contributing separately to depressive cognitions and depressive symptoms.

#### Integrated conceptual model

Building on prior research, the present study proposes a parallel mediation framework to clarify how early adversity and personal strengths relate to depressive outcomes. Specifically, both ACEs and resilience are conceptualised as independent predictors that operate through a common cognitive mechanism – depressive cognitions.

From a cognitive – developmental perspective, exposure to childhood adversity may lead individuals to internalise maladaptive schemas characterised by helplessness, worthlessness, and hopelessness (Beck et al., 1979; Korotana et al., 2016). These depressive cognitions – negative beliefs about the self, world, and future – are known to increase vulnerability to depression (Braet et al., 2015; Briere & Elliott, 1994). Conversely, resilience represents a stable internal capacity that facilitates flexible coping and positive appraisal of stressors (Connor & Davidson, 2003; Wagnild; 1993). Individuals with higher trait resilience are more likely to hold adaptive cognitions – such as self-efficacy, optimism, and hope – which help mitigate depressive cognitions and, in turn, reduce depressive symptoms (Hatami et al., 2019; Mak et al., 2011; Stapleton et al., 2017).

Accordingly, depressive cognitions serve as a shared cognitive pathway through which both risk (ACEs) and protection (resilience) exert their influence on depressive symptoms. Rather than conceptualising resilience as a moderator of ACEs or as a sequential mediator following ACEs, this model posits two distinct yet converging paths: one linking ACEs to depression via heightened depressive cognitions, and another linking resilience to depression via diminished depressive cognitions. This framework integrates cognitive vulnerability and resilience theory, emphasising that depressive cognitions are not only outcomes of early adversity but also mechanisms through which personal resources promote mental health.

Understanding these interrelations is particularly relevant during emerging adulthood, a developmental phase marked by identity exploration, instability, and increasing psychological demands. By jointly examining ACEs, resilience, and depressive cognitions, the current model provides a balanced perspective on risk and protective processes underlying depressive symptoms among university students.

### Research gap and present study

Despite extensive evidence linking ACEs, resilience, depressive cognitions, and depressive symptoms, few studies have examined how these variables operate within a unified framework among university student populations. Most prior research has focused on clinical samples or younger adolescents, leaving a gap in understanding how these relationships function during emerging adulthood – a developmental stage marked by identity exploration and heightened vulnerability to stress.

Moreover, while resilience has consistently been conceptualised as a protective factor, limited research has investigated how resilience and ACEs may each contribute to depressive symptoms through shared cognitive mechanisms. To address these gaps, the present study adopted a parallel mediation framework, in which ACEs and resilience were modelled as independent predictors associated with depressive symptoms both directly and indirectly through depressive cognitions – with ACEs expected to increase depressive cognitions and resilience expected to reduce them.

By testing this framework in a sample of Taiwanese university students, the study aims to clarify how early adverse experiences and personal strengths jointly relate to depressive outcomes through cognitive processes, thereby offering a more comprehensive understanding of risk and protection during emerging adulthood.

### Aims of the study and hypotheses

The present study adopted a parallel mediation framework to examine how adverse childhood experiences (ACEs) and resilience independently relate to depressive symptoms both directly and indirectly through depressive cognitions. Specifically, this study aimed to determine (1) whether greater ACE exposure predicts higher levels of depressive cognitions and depressive symptoms, and (2) whether higher resilience predicts lower levels of depressive cognitions and depressive symptoms.

Based on theoretical and empirical evidence, four directional hypotheses were proposed:

1. ACEs will be positively associated with depressive symptoms, and this association will operate in part through higher levels of depressive cognitions.
2. Resilience will be negatively associated with depressive symptoms, and this association will operate in part through lower levels of depressive cognitions.
3. Higher ACE exposure will predict greater depressive cognitions, which will in turn be associated with higher depressive symptom levels.
4. Higher resilience will predict lower depressive cognitions, which will in turn be associated with lower depressive symptom levels.

By specifying both the direct and indirect pathways, this study aims to clarify how early adversity and personal psychological resources jointly influence depressive symptoms among university students, thereby informing interventions that focus on cognitive restructuring and that help students draw upon or strengthen the functional use of their resilience-related capacities.

## Materials and methods

### Study design

This study utilised a cross-sectional correlational survey design to examine the associations among ACEs, depressive cognitions, resilience, and depressive symptoms in university students.

### Participants and procedures

The present study examined ACEs and related psychological factors among university students in Taipei City and New Taipei City in northern Taiwan. An online questionnaire was developed using Google Forms, which included measures of demographic characteristics, ACEs, resilience, depressive cognitions, and depressive symptoms. All demographic information and psychological responses were self-reported by participants through the online survey platform.

Participants aged 20 or older and enrolled in full-time undergraduate programs from five universities were eligible. Data collection was conducted in two steps. First, the researcher contacted instructors who agreed to allow survey administration during class. Second, the researcher attended the class to explain the study’s purpose and procedures, after which students were invited to participate. Those who agreed provided informed consent electronically and completed the anonymous online questionnaire, which took approximately 15–20 minutes to finish. Upon completion, each participant received a convenience store gift card valued at NT$50 (approximately USD $1.67).

Between March and April 2022, a total of 562 students participated. Ten respondents (1.8%) were excluded due to invalid responses, such as selecting identical options across all items. The final analytic sample consisted of 552 valid participants.

### Measures

#### Adverse childhood experiences (ACEs)

ACEs were assessed using the Chinese version of the Adverse Childhood Experience International Questionnaire (C-ACEs-IQ) (Ho et al., 2019), a retrospective self-report measure evaluating childhood adversities before age 18. The questionnaire includes 12 categories of ACEs (e.g., abuse, neglect, family dysfunction, and violence) using 25 items, excluding collective violence due to its rarity in Taiwan (Kim, 2017; Tran et al., 2015).

Scoring followed the frequency method (Ho et al., 2019), ensuring alignment with international norms while adjusting for cultural differences in emotional neglect and physical abuse criteria. Each endorsed ACEs category was assigned a score of one, with total scores reflecting the number of ACEs categories experienced. Higher scores indicate greater childhood adversity exposure. The Cronbach’s alpha for the C-ACEs-IQ in this study was 0.61, indicating relatively low internal consistency.

#### Resilience

Resilience was assessed using the Connor-Davidson Resilience Scale (CD-RISC; Connor & Davidson, 2003), a 25-item measure evaluating personal resilience traits and adaptive capacities under stress. Items are rated on a 5-point Likert scale (0–4), with total scores ranging from 0 to 100, where higher scores indicate greater resilience. The CD-RISC has been widely validated in university student populations (Dong et al., 2021; Peng et al., 2017). The Cronbach’s alpha in this study was 0.94, demonstrating excellent internal consistency.

Consistent with Connor and Davidson’s (2003) conceptualisation, resilience in this study was regarded as a relatively stable personal trait that reflects an enduring capacity to adapt to stress and recover from adversity, rather than a transient emotional state. Empirical evidence supports this interpretation: the 25-item CD-RISC has demonstrated excellent test – retest reliability (intraclass correlation coefficient = .93; Skaldere-Darmudasa & Sudraba, 2023), and longitudinal data using the 10-item version have shown substantial temporal stability over 1.5- to 2-year intervals (*r* = .66 –.71; Didriksen et al., 2025). These findings indicate that the CD-RISC captures a relatively stable, trait-like dimension of resilience whose core capacity remains consistent across time. Although early adverse experiences may influence the overall level of resilience developed across the life course, the CD-RISC-25 measures an enduring personal capacity for adaptation that is conceptually distinct from transient reactions to past adversity.

#### Depressive cognitions

Depressive cognitions were assessed using the Chinese version of the Cognitive Triad Inventory (C-CTI), adapted from the original CTI (Beckham et al., 1986), which evaluates negative views of the self, world, and future (Beck et al., 1979). The C-CTI consists of 15 items, refined from the original 36-item version through factor analysis. Responses are rated on a 7-point Likert scale, with total scores ranging from 15 to 105; higher scores indicate more negative cognitive patterns, while lower scores suggest a more positive outlook. The Cronbach’s alpha in this study was 0.92, demonstrating excellent internal consistency.

#### Depressive symptoms

Depressive symptoms was assessed using the Chinese version of the Center for Epidemiologic Studies Depression Scale (C-CES-D), translated from the original CES-D (Radloff, 1977) by Chien and Cheng (1985). This 20-item self-report scale, widely used in Taiwan, employs a 4-point Likert scale with total scores ranging from 0 to 60, where higher scores indicate greater depressive symptomatolog. The Cronbach’s alpha in this study was 0.91, demonstrating excellent internal consistency.

#### Demographic data

Demographic data collected in this study included gender, age, study grade, father’s and mother’s education level, and household financial status.

Caregiver education was assessed by asking participants to indicate the highest educational attainment of their father and mother. Responses were categorised into four groups: (1) middle school or below, (2) high school or vocational school, (3) junior college, and (4) university or above.

Household financial status was assessed using a self-rated single item asking participants to evaluate their family’s economic condition. Responses were grouped into three categories: (1) less affluent, (2) intermediate, and (3) more affluent.

#### Statistical analysis

Data were analysed using SPSS 25.0 for descriptive statistics and Rstudio (lavaan package) for structural equation modelling (SEM). Pearson correlation coefficients were calculated to examine associations among study variables. SEM was applied to test the hypothesised path model, analysing both direct and indirect effects of ACEs, resilience, and depressive cognitions on depressive symptoms. Since the model was fully saturated (χ^2^ = 0, df = 0), model fit indices were not applicable. The maximum likelihood (ML) estimation method was used for parameter estimation. Bootstrapping with 1000 resamples was conducted to assess the significance of indirect effects. All statistical tests were two-tailed, with *p*-values < .05 considered statistically significant.

In the hypothesised structural model, both ACEs and resilience were treated as exogenous variables that simultaneously predicted depressive cognitions and depressive symptoms. Depressive cognitions were modelled as an indirect pathway linking ACEs and resilience to depressive symptoms, which served as the final outcome variable.

Covariates. Demographic variables (e.g., age, gender, study grade, and parental education) were examined in preliminary analyses but were not significantly correlated with the main study variables. Given the relative homogeneity of the sample in terms of age and education level, and the theoretical focus on the psychological mechanisms among ACEs, resilience, depressive cognitions, and depressive symptoms, no covariates were included in the final SEM model.

## Results

### Participant characteristics

A total of 552 university students participated in this study. The demographic characteristics of the sample are presented in Table 1. The sample consisted of 54.7% males (*n* = 302) and 45.3% females (*n* = 250). The majority of participants were aged 20 (35.0%) or 21 (41.3%) years. In terms of academic year, most students were in their senior year (47.3%), followed by juniors (34.6%), sophomores (12.9%), and a small percentage in a five-year program (5.3%).

**Table 1.** Participant characteristics.

Variable	n	%
Gender		
Male	302	54.7
Female	250	45.3
Age		
20	193	35.0
21	228	41.3
22	106	19.2
23	25	4.5
Study grade		
Sophomore	71	12.9
Junior	191	34.6
Senior	261	47.3
Grade five	29	5.3
Father’s education level		
≤ Middle school	64	11.6
High School or vocational school	187	33.9
Junior university	114	20.7
university+	187	33.9
Mother’s education level		
≤ Middle school	57	10.3
High School or vocational school	219	39.7
Junior university	128	23.2
University+	148	26.8
Household financial status		
Less affluent	184	33.3
Intermediate	318	57.6
More affluent	50	9.1

*Note: N* = 552.

Regarding parental education, 33.9% of fathers and 26.8% of mothers had a university degree or above, while a substantial portion had completed high school or vocational school (33.9% of fathers and 39.7% of mothers). Fewer parents had attained junior college or middle school or below education levels. Concerning household financial status, 33.3% of students reported less affluent backgrounds, 57.6% reported intermediate financial status, and 9.1% identified as more affluent.

Regarding ACEs, 252 participants (45.7%) reported experiencing at least one ACE, whereas 300 participants (54.3%) reported none. Among those with ACE exposure, most reported experiencing one or two categories of adversity (35.3%), while 5.1% reported three categories and 5.3% reported four or more ACEs. Overall, nearly half of the university students had experienced at least one category of childhood adversity.

In addition to overall ACE exposure, the distribution of ACE categories was examined. ACEs related to child maltreatment were most common (43.7%), followed by household dysfunction (37.6%), whereas exposure to violence outside the home was least frequently reported (4.5%). At the individual category level, the most frequently endorsed ACEs were emotional neglect (15.2%), parental separation, divorce, or absence of one or both parents (14.5%), witnessing domestic violence (12.1%), and physical abuse (11.6%). In contrast, witnessing community violence (1.6%) and having a household member incarcerated (1.8%) were the least frequently reported categories.

In terms of depressive cognitions, 295 participants (53.4%) scored above the sample mean on the Depressive Cognitions Scale, indicating a moderate to high level of negative cognitive patterns within the sample.

Descriptive statistics and analyses of depressive symptoms are presented in the following section.

### Descriptive statistics and correlations

The means, standard deviations, and correlations among study variables are presented in Table 2. The average ACEs score was 0.86 (*SD* = 1.32), indicating a relatively low level of reported adverse experiences in childhood. The mean resilience score was 57.21 (*SD* = 15.79), while the mean depressive cognitions score was 50.87 (*SD* = 16.06). The mean depressive symptoms score (CES-D) was 17.23 (*SD* = 9.68).

**Table 2.** Means, standard deviations, and correlations among study variables.

Variable	Means	SD	1.	2.	3.	4.
1.ACEs	0.86	1.32	1			
2.Resilience	57.21	15.79	−.208**	1		
3.Depressive cognitions	50.87	16.06	.302**	−.716**	1	
4.Depressive symptoms	17.23	9.68	.330**	−.693**	.730**	1

*Note: N* = 552.

**p < .01.

According to the validation study of the Chinese version of the CES-D (Chien & Cheng, 1985), a total score of 16 or higher indicates clinically meaningful depressive symptoms, with good sensitivity (92%) and specificity (91%). The present sample’s mean score exceeded this threshold, suggesting that, on average, participants reported mild to moderate levels of depressive symptoms, and that depressive mood was relatively prevalent among university students.

Pearson correlation analyses revealed several significant relationships among the study variables: ACEs were positively correlated with depressive cognitions (*r* = .302, *p* < .01) and depressive symptoms (*r* = .330, *p* < .01). Resilience was negatively correlated with depressive cognitions (*r* = −.716, *p* < .01) and depressive symptoms (*r* = −.693, *p* < .01). Depressive cognitions was strongly associated with depressive symptoms (*r* = .730, *p* < .01). These findings suggest that ACEs are associated with higher depressive cognitions and depressive symptoms, while resilience appears to have a protective role.

### Path analysis

#### Direct and indirect effects

**Figure 1.**
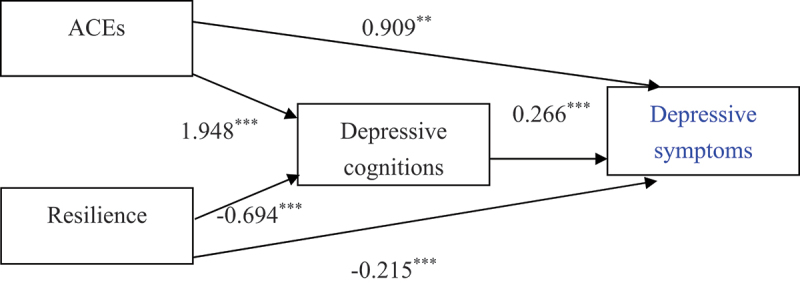
Structural equation model of the relationships between ACEs, resilience, depressive cognitions, and depressive symptoms among university students.

*Note*. This path model illustrates both the direct and indirect effects of ACEs, resilience, and depressive cognitions on depressive symptoms. ACEs and resilience are treated as exogenous variables; depressive cognitions as the mediator; and depressive symptoms as the final outcome. All path coefficients are unstandardised (*b* coefficients).

A structural equation modelling (SEM) analysis was conducted to examine the direct and indirect effects of ACEs, resilience, and depressive cognitions on depressive symptoms. The results of the path analysis are presented in Table 3 and Figure 1.

ACEs = Adverse Childhood Experiences measured by the C-ACEs-IQ; Depressive Cognitions = Cognitive Triad Inventory (C-CTI); Resilience = Connor-Davidson Resilience Scale (CD-RISC-25); Depressive Symptoms = Center for Epidemiologic Studies Depression Scale (C-CES-D).

**p < .01, ***p < .001.

**Table 3.** The direct and indirect effects of path model.

Effect variable	Variable	Variable	*b*	SE	*β*
Indirect effect					
ACEs	Depressive cognitions	Depressive symptoms	0.518***	0.113	0.070
Resilience	Depressive cognitions	Depressive symptoms	−0.185***	0.023	−0.301
Direct effect					
ACEs	Depressive symptoms		0.909**	0.279	0.124
Resilience	Depressive symptoms		−0.215***	0.028	−0.351
Depressive cognitions	Depressive symptoms		0.266***	0.029	0.441
ACEs	Depressive cognitions		1.948***	0.460	0.160
Resilience	Depressive cognitions		−0.694***	0.027	−0.683

**p < .01.

***p < .001.

#### Direct effects

ACEs had a direct positive effect on depressive symptoms (*b* = 0.909, *SE* = 0.279, *β* = 0.124, *p* < .01). Resilience had a direct negative effect on depressive symptoms (*b* = −0.215, *SE* = 0.028, *β* = −0.351, *p* < .001). Depressive cognitions significantly predicted depressive symptoms (*b* = 0.266, *SE* = 0.029, β = 0.441, *p* < .001). ACEs had a significant positive effect on depressive cognitions (*b* = 1.948, *SE* = 0.460, *β* = 0.160, *p* < .001). Resilience had a strong negative effect on depressive cognitions (*b* = −0.694, *SE* = 0.027, *β* = −0.683, *p* < .001).

#### Indirect effects

ACEs had an indirect effect on depressive symptoms through depressive cognitions (*b* = 0.518, *SE* = 0.113, *β* = 0.070, *p* < .001). This indicates that greater exposure to childhood adversity predicted higher levels of depressive cognitions, which in turn were associated with more severe depressive symptoms.

Resilience also showed an indirect effect on depressive symptoms via depressive cognitions (*b* = −0.185, *SE* = 0.023, *β* = −0.301, *p* < .001). This suggests that higher levels of resilience predicted lower depressive cognitions, which were associated with fewer depressive symptoms.

Together, these results suggest that the direct pathways between ACEs, resilience, and depressive symptoms are complemented by significant indirect pathways through depressive cognitions, highlighting the cognitive mechanisms underlying both risk and protection.

#### Summary

These findings indicate that both ACEs and resilience exerted indirect effects on depressive symptoms through depressive cognitions, where greater ACE exposure led to more negative cognitive patterns and consequently higher depressive symptoms, whereas higher resilience was linked to more adaptive cognitions and thus lower depressive symptoms.

The protective role of resilience operates both directly – by reducing depressive symptoms – and indirectly – by attenuating negative cognitive patterns associated with depressive symptoms.

## Discussion

### Correlation between study variables

Consistent with prior research (Braet et al., 2015; Kim, 2017; Stark et al., 1996; Tran et al., 2015; Ying et al., 2014), the correlational results generally supported the hypothesised relationships among ACEs, resilience, depressive cognitions, and depressive symptoms.

Given that these bivariate associations were preliminary and not central to the main research questions, only a brief summary is provided here.

These results suggest that early adverse experiences are linked to greater depressive vulnerability, whereas resilience functions as a protective factor against maladaptive cognitions and depressive symptoms.

### Path analysis of study variables

Structural equation modelling (SEM) results indicated that ACEs were significantly and positively associated with depressive symptoms, even when resilience and depressive cognitions were included in the model. This finding aligns with previous studies showing that greater exposure to ACEs correlates with higher levels of depressive symptoms among university students (Windle et al., 2018) and adulthood (Chang et al., 2019).

Resilience was significantly and negatively associated with depressive symptoms, consistent with findings from studies in both the general population (Ran et al., 2020) and adolescent survivors of traumatic events (Ying et al., 2014). Individuals reporting higher levels of trait resilience – characterised by emotional flexibility and adaptive coping – tended to report fewer depressive symptoms.

Depressive cognitions was also significantly related to depressive symptoms, supporting Beck’s Cognitive Theory of Depression (Beck, 1987), which posits that individuals with negative cognitive biases are more likely to exhibit depressive symptom patterns. The cognitive triad – negative beliefs about the self, the world, and the future – was strongly linked to depressive symptoms, particularly among individuals exposed to early-life adversity (Braet et al., 2015).

### Indirect role of depressive cognitions

This study further examined the indirect role of depressive cognitions in the associations among ACEs, resilience, and depressive symptoms. The results indicated that depressive cognitions had an indirect effect linking ACEs and depressive symptoms, consistent with prior findings suggesting that childhood adversity is associated with maladaptive cognitive patterns, which in turn relate to greater depressive symptomatology (Briere & Elliott, 1994; Korotana et al., 2016; Sinclair et al., 2012).

Specifically, greater exposure to ACEs was associated with more depressive cognitions about the self, world, and future, which contributed to higher depressive symptoms. The indirect effect (*b* = 0.518, *p* < .001) was smaller than the direct effect (*b* = 0.909, *p* < .001), suggesting that while depressive cognitions represents an important psychological correlate in the association between ACEs and depressive symptoms, ACEs themselves remain directly related to depressive symptoms. These results align with prior literature emphasising the societal importance of preventing ACEs, given their established associations with long-term psychological consequences.

Similarly, depressive cognitions was found to have an indirect association between resilience and depressive symptoms. Higher resilience predicted fewer depressive cognitions, which in turn were associated with lower depressive symptoms. This finding aligns with previous research indicating that individuals with higher resilience tend to exhibit adaptive characteristics, such as goal-setting, confidence, adaptability, and social problem-solving skills, which help counteract negative cognitive patterns (Connor & Davidson, 2003; Li & Zheng, 2025; Stapleton et al., 2017). The indirect effect of resilience on depressive symptoms through depressive cognitions (*b* = −0.185, *p* < .001) was slightly smaller than its direct effect (*b* = −0.215, *p* < .001), suggesting that resilience may be primarily related to lower levels of depressive symptoms, while also exerting a secondary protective effect by mitigating negative cognitive styles.

### Implications for practice

The findings of this study have important implications for psychological service providers working with university students. Although causal relationships cannot be established from this cross-sectional design, the observed associations underscore the importance of early identification and psychological support for students with a history of ACEs. Screening tools for ACEs and depressive cognitions could be considered for integration into university counselling services to help identify at-risk students, rather than implying causal prevention.

Furthermore, previous research suggests that cognitive-behavioural approaches may be effective in addressing maladaptive thought patterns associated with depressive symptoms. Cognitive restructuring and trauma-informed education could therefore be explored in future longitudinal or intervention research (Oral et al., 2016). Programs that support students in strengthening adaptive coping resources may also be explored in future research to assist those with ACE-related psychological distress.

Preventing ACEs remains an important public health priority. While this study cannot determine causal effects, prior research has underscored the value of promoting positive parenting practices, increasing access to mental health resources, and implementing policies that support children’s well-being. These findings collectively suggest directions for policy and prevention, though further longitudinal research is needed to confirm causal mechanisms.

### Depressive symptom levels in context

The mean CES-D score in this study was 17.23 (SD = 9.68), exceeding the clinical cut-off of 16 for the Chinese version (Chien & Cheng, 1985) and indicating mild to moderate depressive symptoms among university students. When compared with the findings of Chang and Chen (2018), who used the same CES-D scale to assess depressive symptoms among design freshmen in southern Taiwan (*N* = 120) and reported a higher mean score of 24.8, the present sample demonstrated a relatively lower average level of depressive symptoms. This difference may reflect variations in participants’ academic disciplines, year levels, or adjustment experiences, as design students often encounter intensive creative workloads and irregular study schedules that may heighten emotional distress. Overall, the results suggest that while depressive symptoms were common in this university population, their severity appeared lower than that reported in certain high-stress academic contexts.

### Strengths, limitations, and future directions

This study contributes to the existing literature by providing a comprehensive analysis of the relationships among ACEs, resilience, depressive cognitions, and depressive symptoms in a sample of university students. The inclusion of resilience as both a direct and indirect protective factor offers a broader perspective on its potential role in psychological adaptation following early-life adversity, without implying a confirmed causal mechanism.

However, several limitations must be acknowledged. First, the retrospective self-report nature of the ACEs measure may introduce recall bias. Second, because this study employed a cross-sectional design, causal relationships cannot be inferred, and all findings should be interpreted as correlational rather than causal. Future research should therefore use longitudinal or experimental designs to clarify the temporal sequence and potential causal pathways among these variables. Third, while this study assumes that ACEs before age 18 are related to current depressive symptoms, some participants may have experienced depressive symptoms in the past and later recovered, further complicating causal interpretation.

In addition, the internal consistency of the C-ACEs-IQ was relatively low, which may reflect the multidimensional and heterogeneous nature of ACEs. This limitation should be taken into account when interpreting the findings.

Additionally, other unmeasured variables such as academic stress, social support, and personality traits may influence the observed associations. Future research incorporating these factors would help build a more comprehensive model of depressive symptoms among university students.

Finally, the generalisability of the findings should be interpreted with caution. Although the sample size was adequate, participants were recruited from five universities in northern Taiwan using convenience sampling. Therefore, the results may not generalise to students from other universities, regions, or educational systems within Taiwan, nor to populations in other cultural contexts. Future studies should aim to include more diverse samples across geographic and institutional settings to enhance external validity.

Overall, depressive cognitions emerged as an important psychological correlate between ACEs, resilience, and depressive symptoms. These findings provide a valuable empirical foundation for future research and preventive efforts aimed at reducing depressive vulnerability in emerging adults, while recognising that causal inferences cannot be drawn from the present design.

## Conclusion

The findings of this preliminary study suggest that higher ACEs, lower resilience, and greater depressive cognitions are closely associated with depressive symptoms among university students in northern Taiwan. Depressive cognitions appeared to function as a potential indirect pathway linking ACEs and resilience to depressive symptoms. These findings underscore the importance of considering early adverse experiences, cognitive patterns, and resilience when addressing mental health concerns in emerging adults. Future research may further explore how approaches targeting cognitive processes and broader adaptive resources relate to depressive symptoms in university populations, while acknowledging that causal relationships cannot be inferred from this design.

## Data Availability

The data that support the findings of this study are available from the corresponding author upon reasonable request.
